# Immobilization of FGF on Poly(xylitol dodecanedioic Acid) Polymer for Tissue Regeneration

**DOI:** 10.1038/s41598-020-67261-6

**Published:** 2020-06-26

**Authors:** Negar Firoozi, Yunqing Kang

**Affiliations:** 1 Department of Ocean & Mechanical Engineering, Florida Atlantic University, 777 Glades Road, Boca Raton, Florida 33431 United States; 2 Department of Biomedical Science, Florida Atlantic University, 777 Glades Road, Boca Raton, Florida 33431 United States; 3 Integrative Biology Ph.D. Program, Department of Biological Science, Florida Atlantic University, 777 Glades Road, Boca Raton, Florida 33431 United States

**Keywords:** Biotechnology, Biomaterials, Drug delivery

## Abstract

Fibroblast growth factor (FGF) plays a vital role in the repair and regeneration of most tissues. However, its low stability, short half-life, and rapid inactivation by enzymes in physiological conditions affect their clinical applications. Therefore, to increase the effectiveness of growth factors and to improve tissue regeneration, we developed an elastic polymeric material poly(xylitol dodecanedioic acid) (PXDDA) and loaded FGF on the PXDDA for sustained drug delivery. In this study, we used a simple dopamine coating method to load FGF on the surface of PXDDA polymeric films. The polydopamine-coated FGF-loaded PXDDA samples were then characterized using FTIR and XRD. The *in vitro* drug release profile of FGF from PXDDA film and cell growth behavior were measured. Results showed that the polydopamine layer coated on the surface of the PXDDA film enhanced the immobilization of FGF and controlled its sustained release. Human fibroblast cells attachment and proliferation on FGF-immobilized PXDDA films were much higher than the other groups without coatings or FGF loading. Based on our results, the surface modification procedure with immobilizing growth factors shows excellent application potential in tissue regeneration.

## Introduction

Wound represents a significant health challenge. Chronic wounds (or skin ulcers) account for approximately 37 million skin wounds globally and 6 million skin wounds in the United States^1^. Skin grafting is an intervention for wound healing, although it still has many drawbacks in the repair and regeneration of the skin tissue. Over the past decades, tissue engineering has been brought up as a promising option for traditional therapies by combining engineering materials, cells, and suitable biochemical factors. So, one of these promising new areas of tissue engineering is the treatment of chronic wounds using biomaterials and growth factors^2^. The value of these materials is that they replicate the microenvironment and activate crucial regenerative mechanisms conducive to wound healing^3^. Previous research has demonstrated that modulating the release of growth factors from pharmaceutical formulations based on sustained drug delivery system strategies can up-regulate and enhance wound healing, as well as skin regeneration^4–10^.

Growth factors (GFs) are water-soluble proteins and have the ability to regulate many cellular processes. Growth factors are critically crucial for coordinating both cell-cell and cell-matrix interactions during normal injury repair^11–14^. Inadequate growth factor’s bioavailability because of depauperate synthesis or exceeding degradation, is a common characteristic of chronic wounds. Thus, the prospect of exogenous growth factors being efficiently delivered to non-healing wounds may simplify cellular responses and lead to seasonable wound healing^11,12,15,16^. In spite of many promising pieces of research in animal models indicating the enhancement in the speed of healing with the addition of different kinds of growth factors, clinical uses are still narrow as it is challenging to maintain the sustained release of a growth factor. Complex systems developed for the purpose of controlling the spatiotemporal delivery of growth factors are a necessity for the effective and safe use of growth factors as regenerative treatments in clinical application, such as biomaterial-based drug delivery systems^4,17–20^. Therefore, this work studied the effect of fibroblast growth factor (FGF) on cell survival and growth as a purpose of promoting wound healing and decreasing scar in the skin tissue. FGF is a critical growth factor that is representative of the types of GF’s associated with the repair and regeneration of different damaged tissues. FGF was initially being known as a protein that is able to promote the proliferation of fibroblast cells^21,22^. Then, FGFs potential biological functions were making them capable of being applicable for repair and regeneration of various tissues such as skin, bone, tendon, muscle, nerve, tooth, blood vessel, cartilage, and adipose^13,23–26^.

Biomaterials are capable of serving as a supporting structure intended for facilitating cell growth and differentiation, also functioning as a platform for growth factor delivery that can potentially promote more efficient wound healing. Growth factors can be loaded onto biomaterial scaffolds by physical adsorption, encapsulation during the scaffold preparation processes, such as phase separation, particulate leaching, and solvent casting, gas foaming, melt molding, and freeze-drying. The scaffold that is loaded with a growth factor can then be implanted at the wound site in order to protect the tissue damage and enhance wound healing^5,8,27–32^. Over the last twenty years, much attention has been placed on the physical immobilization of growth factors onto different biomaterials, because it is easy to process under moderate conditions at room temperature. However, it is challenging for these methods to load growth factors without comprising its bioactivities and effectively control its release. Recent studies were using the reactivity of polydopamine layers in order to immobilize GFs onto many different matrices^16,30^. The dopamine coating and loading method is a mild and facile method to load GFs effectively and release them sustainably, which can ultimately help tissue repair and regeneration. Polydopamine mostly haltered GFs onto biomaterials surfaces by covalent bonding between the quinone groups of polydopamine (oxidation of catechol groups in dopamine) and of GFs’ amino groups trough Schiff-base reactions and Michael-type addition reactions^27,29,30,33,34^.

In our previous study, we synthesized a new, elastic, biocompatible, and biodegradable xylitol-based polymer poly(xylitol-dodecanedioic acid) (PXDDA)^35^. This xylitol-based polymer was synthesized by a melt condensation method that we developed. The method does not need any toxic catalyst, and it is quick, simple, and cost-effective without any addition of other chemicals^36,37^. In the study, we synthesized different kinds of xylitol-based polymers with various stoichiometric ratios of xylitol and dodecanedioic acid, and also characterized their physicochemical and biological properties based on other related studies^35,38,39^. Our synthesized PXDDA polymer showed different elasticities. The elasticities vary with the ratios of xylitol to dodecanedioic acid or curing time. Compared with the common polymer polylactic acid (PLA)^40^, PXDDA also exhibited exceptional biocompatibility *in vitro*. Furthermore, the PXDDA polymer demonstrated a new auto-fluorescent property^35^.

Based on our developed PXDDA polymer, in this study, we further developed FGF immobilized PXDDA films inspired by polydopamine, that could be used as substrates for guided tissue regeneration. We used a straightforward method under a mild condition to coat the PXDDA surface with polydopamine and immobilize fibroblast growth factor, in order to study their effect on the *in vitro* cell behavior. Fourier transform infrared spectroscopy (FTIR) and X-ray diffraction (XRD) analyses of the films confirmed the successful deposition of the polydopamine on the PXDDA surface. We investigated the effect of polydopamine coating on FGF immobilization and release, and we also examined the cellular responses on the PXDDA films with/without coating. The results demonstrated successful immobilization of FGF by polydopamine and improved cell viability.

## Materials and Methods

Dopamine hydrochloride, 99%, was purchased from Alfa Aesar. Dimethyl sulfoxide (DMSO) and 3-(4,5-dimethylthiazol-2yl)-2,5diphenyl tetrazolium bromide (MTT) were purchased from Sigma. Recombinant Mouse FGF basic and Mouse/Rat FGF basic Quantikine ELISA Kit were purchased from R&D Systems. BCA Protein Assay Kit was purchased from Abcam. All other commercially available solvents were obtained from Fisher Scientific Company.

### Preparation of (Dopamine-HCL)-Coated PXDDA Discs

We prepared the dopamine solution by dissolving 4 mg of dopamine in one ml of 10 mM Tris-HCl buffer solution (pH 8.5). For coating, PXDDA discs were immediately immersed into a dopamine hydrochloride solution at 37 °C, and the solution was gently shaken up to 24 h at 100 rpm. At each time point, PXDDA discs were washed entirely in deionized water for 1 h to remove the extra amount of dopamine that did not react. Following, the coated PXDDA discs were dried in the oven for 24 h at 40 °C^29,30,33^.

The polydopamine-coated PXDDA samples were characterized by Fourier-transform infrared spectroscopy (FTIR; Jasco FT/IR-4100) and X-ray powder diffraction (XRD; Siemens D5000 X-ray Diffractometer with monochromatic Cu Kα radiation, a 40 kV accelerating voltage, a current of 30 mA, and scanning in the 2θ range of 10° to 60° with a step size of 0.05) to confirm the dopamine layers deposition. Dopamine on PXDDA discs was also quantified using the BCA assay. The amount of coated dopamine on the surface of the PXDDA discs was the difference between the concentrations of dopamine before coating and after coating.

The coated discs of 8 mm diameter were treated with 300 μL BCA working reagent and incubated at 37 °C for 120 min. For detecting the concentration of BCA, we used a spectrometer to determine the absorbance at 562 nm (SpectraMax 190 Microplate Reader)^29,30^.

### Immobilization and Release of Fibroblast Growth Factor

Polydopamine-coated PXDDA discs were immersed in 800 μL of FGF solutions prepared by dissolving different amounts of FGF (250 and 500 ng) in a 10 mM Tris-HCl buffer (1 ml, pH 8.5) and incubated at 37 °C for around 24 h with gentle shaking. The discs then were rinsed with water twice. Immobilized amount of FGF was detected using enzyme-linked immunosorbent assay (ELISA). The final immobilized FGF was measured by subtracting the amount of FGF in the supernatant of the Tris-HCl buffer solution from the initial amount in the original solution. Here, dopamine-coated PXDDA and non-coated PXDDA were used to immobilize FGF through the chemical bonding (coated) or physical absorption (non-coated).

The release kinetics of FGF from the discs were also studied in the phosphate-buffered saline (PBS) with the ELISA kit. PXDDA discs were immersed in the 1.5 ml of PBS solution and placed on a shaker at 37 °C. At each interval, 500 μL of the supernatant’s release medium was collected and freshened with an equal amount of PBS on days 3, 7, 14, 21, and 28. Supernatants from each sample were stored at −80 °C until day 28 that we collected all the samples and did the ELISA experiment, using the manufacturer’s protocol. We measured the absorbance of the samples with a spectrophotometer at 450 nm with 540 nm used for correction of λ (SpectraMax 190 Microplate Reader). The tests were repeated three times.

### Human Fibroblast Cells Attachment and Proliferation

Cellular behaviors on the coated PXDDA discs were studied using human primary esophageal fibroblast cells that were purchased from ScienCell Research Laboratories (CA, USA). The polydopamine-coated PXDDA discs were placed into 24-well plates. Discs were sterilized with 70% ethanol for 30 min and then washed thoroughly with PBS. Following, sterilized PXDDA discs were immobilized with FGF by immersing in a FGF solution (500 ng/mL, 10 mM Tris-HCl buffer, pH 8.5) and then samples were incubated overnight at 37 °C. Human fibroblast cells were then seeded onto the PXDDA discs at 10^5^ cells/cm^2^ using Dulbecco’s Modified Eagle Medium (DMEM, Lonza) supplemented with 10% fetal bovine serum (Gibco) and 1% PSG (Penicillin-Streptomycin-Glutamine, Gibco).

Cell proliferation of fibroblast on different groups of PXDDA discs (PXDDA, polydopamine-coated PXDDA, growth factor-immobilized PXDDA) was determined using the 3-(4,5-dimethyl-2-thiazolyl)-2,5-diphenyl tetrazolium bromide (MTT) assay. At each time point, 80 μL of MTT solution (5 mg/mL in PBS) was added into each well, containing 720 μL of culture medium, and incubated at 37 °C for 4 h. Once completed, we removed the medium and added 800 μL of DMSO to each well. After 10 minutes, the absorbance at 570 nm was read using a microplate reader (SpectraMax 190 Microplate Reader).

To study the effect of dopamine and FGF on cell attachment and spreading, the morphology of fibroblast cells cultured on different discs was observed after 3 and 7 days of culture using a scanning electron microscope (SEM; JEOL JSM-6330F, Tokyo, Japan). PXDDA discs were treated with a 2.5% glutaraldehyde solution in PBS for 2 h, followed by dehydration in ethanol 25–100%.

### Statistical analysis

We used one-way ANOVA with Tukey’s post hoc test to compare the groups. All quantitative data are demonstrated as a mean ± standard error (SE) with three samples for different groups. The values of p < 0.05 are considered statistically significant.

## Results

### Characterization of Polydopamine-Coated PXDDA

**Figure 1:**
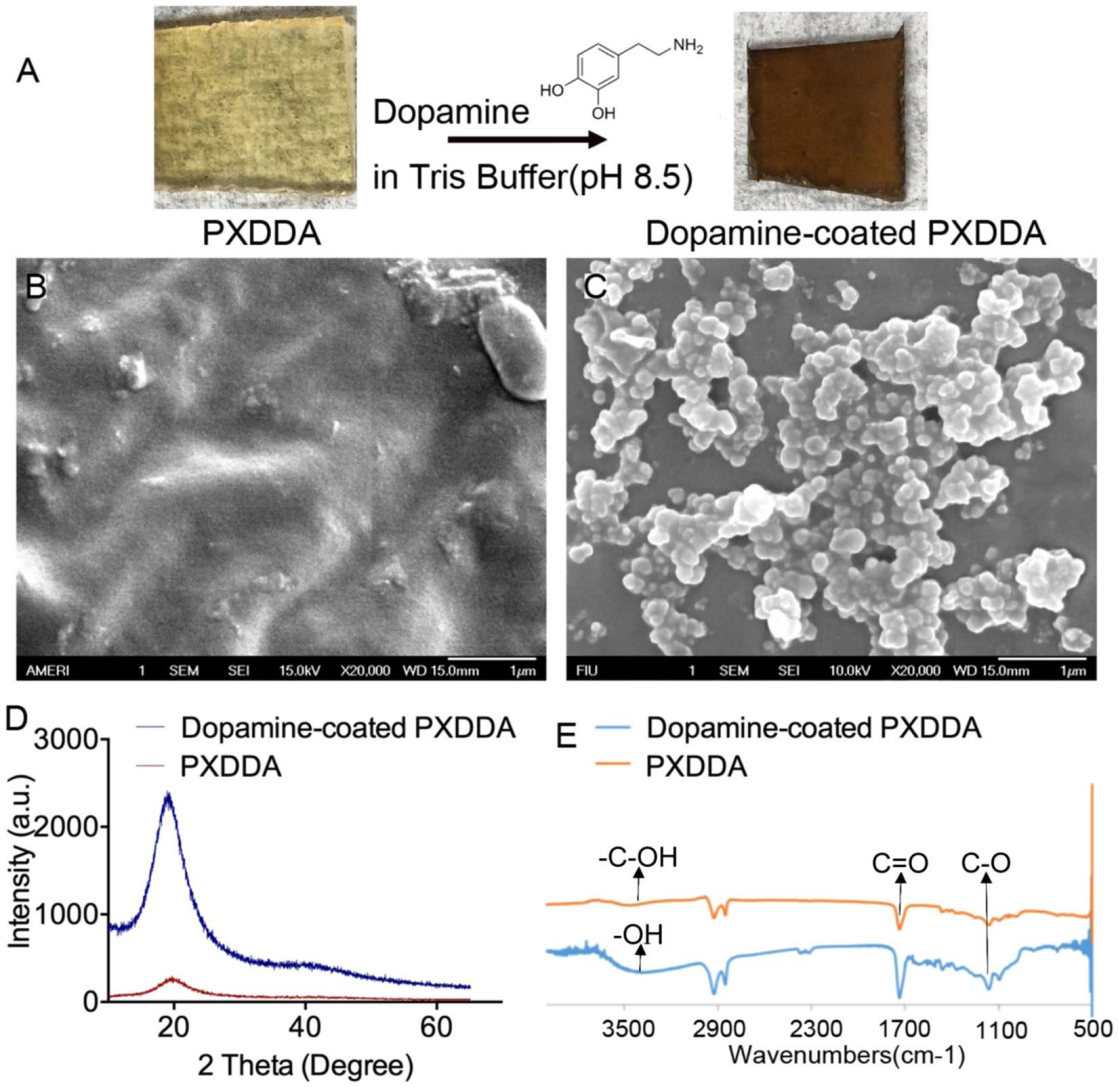
Polydopamine coating process (**A**). SEM images of the surface morphologies of the uncoated PXDDA (**B**) and coated PXDDA with polydopamine (**C**). XRD patterns of PXDDA before and after polydopamine coating (**D**). FTIR spectra of polydopamine-coated PXDDA polymer (**E**).

After the PXDDA was coated by dopamine at pH 8.5 for 24 h, the surface changed from brown to dark (Fig. 1A). The morphology and microstructure of the PXDDA with polydopamine coating was also observed using a SEM. SEM images show that the surface of the PXDDA material is smooth before dopamine coating (Fig. 1B). After coating, the polydopamine-coated PXDDA films showed different surface morphologies. Many clustered polydopamine precipitate particles covered the surface of the materials completely, and the surface became rough (Fig. 1C).

To identify the coating of dopamine on the surface, XRD was performed. The XRD pattern for the polydopamine-coated PXDDA in Fig. 1D shows that the peak at 19.58° became sharp after dopamine coating, compared to the pure PXDDA. The increased broad reflection peak at 19.58° could be due to the polydopamine coating on the PXDDA surface^41^. FTIR spectroscopy was used to study the chemical structure of the coated PXDDA surface. According to Fig. 1E, the absorption band at 3456 cm^−1^, which attributes to the stretching vibrations of –OH groups in the PXDDA, broadened and underwent a small shift to 3392 cm^−1^ for polydopamine-coated PXDDA. Such characteristics are corresponded to the aromatic – C-OH bonding in dopamine. The sharp peaks at 1728 cm^−1^ and 1159 cm^−1^ are also assigned to the C‖O and C–O bonds, respectively. The spectra show that polydopamine-coated PXDDA has similar well-defined characteristic bonds to that in the uncoated PXDDA, but the relative intensities of these characteristic peaks are a little higher, which means that the dopamine coating could have similar functions groups to PXDDA^42^.

**Figure 2:**
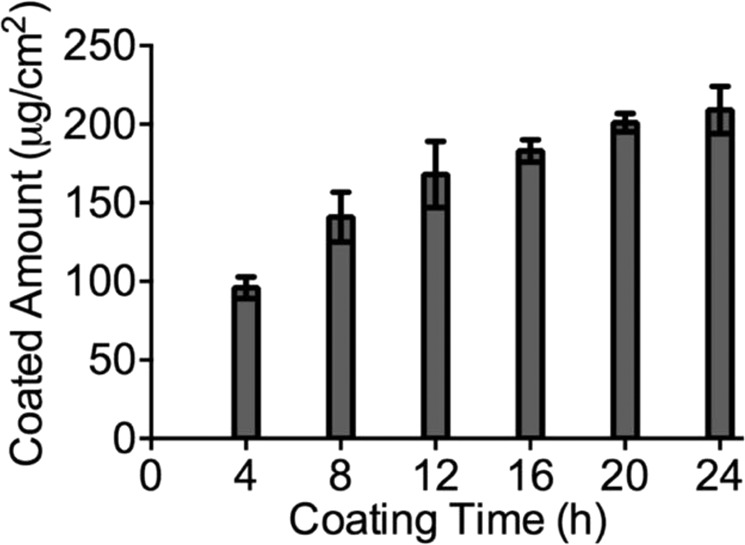
Amounts of polydopamine coated on the PXDDA discs determined using BCA assay.

According to BCA assay results (Fig. 2), the amount of dopamine on the PXDDA surface increased from 96 to 183 μg/cm^2^ as coating time increased from 4 h to 24 h. After 20 h, the coated amount of dopamine did not increase dramatically. It confirmed that the amount of polydopamine coated on PXDDA films is time-dependent before it reached a plateau, which is in 24 h.

### Quantification of Immobilized FGF and Release Profile

**Figure 3:**
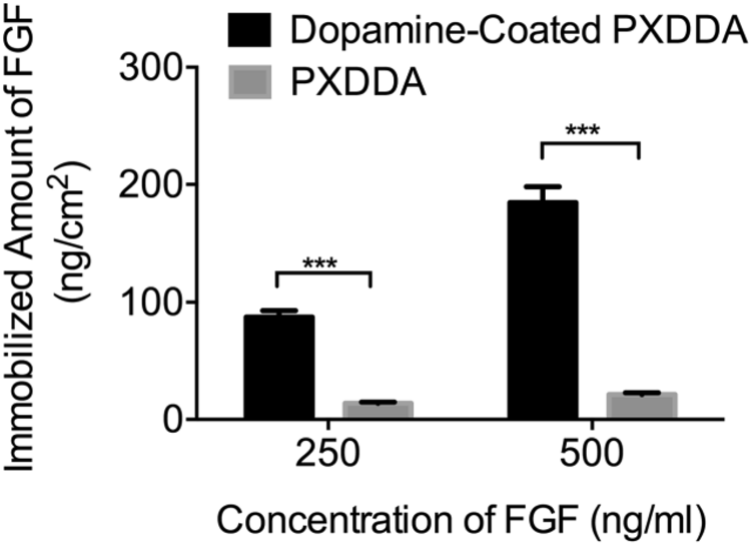
Quantification of FGF immobilized on polydopamine-coated PXDDA discs using ELISA.

The binding efficiency and growth factor release profiles of FGF on polydopamine-coated and uncoated PXDDA discs were defined by ELISA. It can be seen from the result in Fig. 3 that the FGF binding efficiency of the polydopamine-coated disc is higher than that of the uncoated PXDDA disc. The efficiency of FGF immobilization on polydopamine-coated PXDDA discs is approximately 6.2 (250 ng/ml) and 8.5 (500 ng/mL) times higher than that on PXDDA discs without polydopamine coating. The ELISA results also demonstrated that the amount of immobilized growth factor on polydopamine-coated PXDDA increased by increasing the concentration of growth factor in treatment solutions. When 500 μL of FGF with different concentrations of 250 ng/mL and 500 ng/mL were used to treat each cm^2^ of polydopamine-coated discs, the polydopamine-coated group which was treated with 500 ng/mL of FGF, demonstrated more immobilized FGF (184.67 ng/cm^2^) than the other group (87.33 ng/cm^2^). These results confirmed that the polydopamine coating on the surface of polymeric discs would effectively improve the binding efficiency of growth factors to the surface.

**Figure 4:**
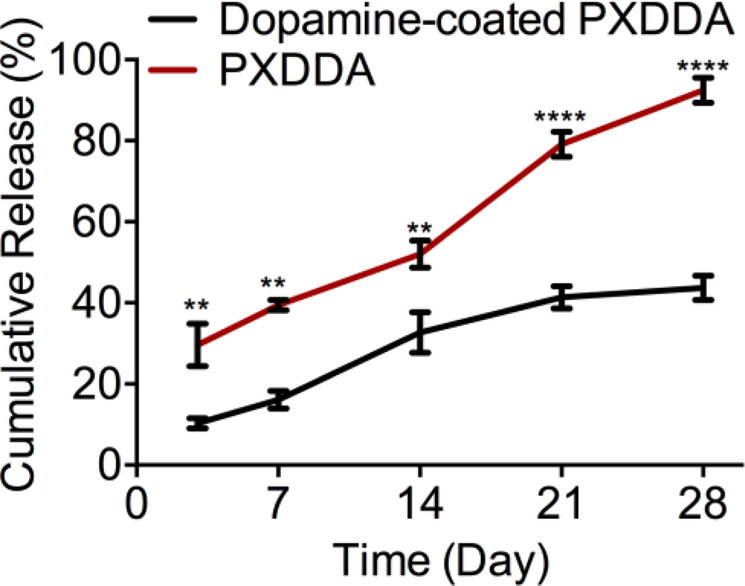
*In vitro* cumulative release profile of FGF.

*In vitro* release profile of growth factor from the grafted surface also indicated that there was a release with 41% and 79% of the total FGF released from the polydopamine-coated and uncoated PXDDA discs within 21 days, respectively (Fig. 4). After 28 days, around 67% of the growth factor was stably retained on the coated PXDDA surface, which confirmed that the polydopamine coating could sustain the *in vitro* release of FGF. The release results showed that the polydopamine coating increased the immobilized growth factors on the PXDDA surface and sustained the release of growth factors. In conclusion, the immobilized growth factors can be released from the dopamine-coated surface in a sustained release way.

### Adhesion and proliferation of fibroblast cells

**Figure 5:**
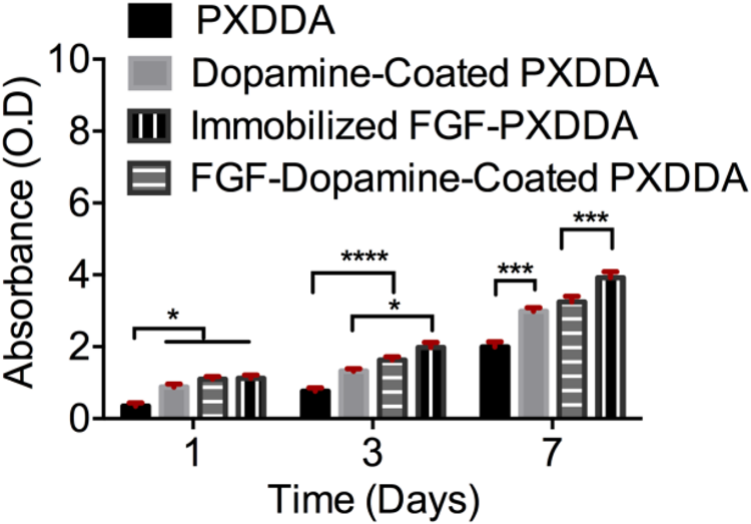
MTT assay results of fibroblast cells culture.

**Figure 6:**
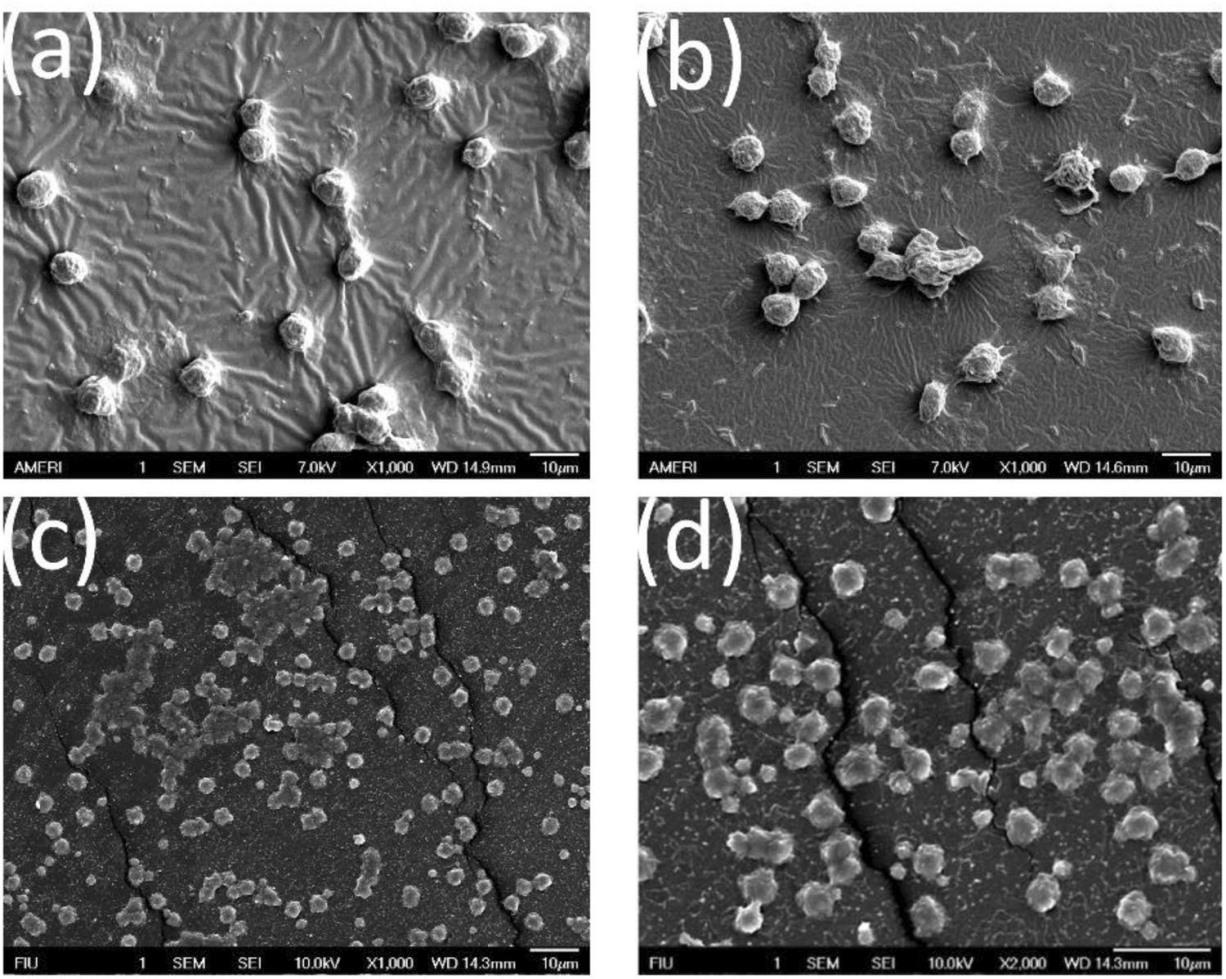
SEM images of fibroblast cells on uncoated PXDDA (**a**), dopamine-coated PXDDA (**b**), FGF-absorbed PXDDA, and FGF-Polydopamine-Coated PXDDA (**d**).

As shown in Fig. 5, the seeded fibroblast cells on the untreated PXDDA discs, indicated the lowest cell proliferation rate with time. After dopamine coating, the cell growth rate was significantly increased in the polydopamine-coated PXDDA discs compared to uncoated PXDDA. As we mentioned earlier, dopamine coating increased the surface roughness, which could have positive effects on cell proliferation. On the other hand, hydroxyl and amine groups of dopamine also promote cell adhesion and proliferation. When the fibroblast cells were cultured on the FGF-immobilized non-coated PXDDA discs, the absorbance significantly increased. This result may be due to the high concentration of FGF released quickly from the surface of PXDDA. The growth factor has an important role in improving tissue regeneration as it increases adhesion and proliferation. These results also confirm that the fibroblast growth factor has improved cell viability. Figure 6 also shows the SEM images of fibroblast cell morphology after 7 days of culture on FGF-polydopamine-coated PXDDA discs. From the SEM, the fibroblast cells showed less cell adhesion and spreading at 7 days on uncoated PXDDA films (Fig. 6a), compared with the dopamine coated PXDDA. After absorbing FGF or immobilizing FGF onto the PXDDA, more cells attached and connected to spread out (Fig. 6c,d).

## Discussion

In this study, we immobilized a growth factor FGF onto a polymer that we developed in our previous study^35^. We found that the dopamine coating can effectively load FGF and control its release.

Over the past twenty years, the physical absorption of growth factors on different biomaterials has become to the attention as it is easy and cost-effective to process under mild conditions^4,14^. However, for physical absorption, the challenge is how to control the sustained release of growth factors. To enhance the loading ability and control release of growth factors, either a new material surface structure is required or a new immobilization method for growth factors is needed. Growth factor delivery systems based on biomaterials (inorganic, natural, or synthetic) with different structures are able to make differential immobilization efficiency and release kinetics in the local environment^43–45^. These new biomaterial systems are still in the preliminary steps of laboratory research and development and need more solid clinical data^46^. An interesting finding in this study is that even the PXDDA without polydopamine coating can also load FGF-2 easily and also can release FGF-2 in a near-linear pattern similar to polydopamine-coated PXDDA. In other words, the slopes of release curves of coating and non-coating are almost the same, but the difference is in the timeline. For example, we have around 40% release for polydopamine-coated PXDDA after 28 days, but it increases to 100% for PXDDA without coating. This result implied that the PXDDA has the ability to absorb the FGF, so the FGF solution went inside the internal pores and after solvent evaporation, the particles stayed there. When released, the FGF gradually released out in an almost linear pattern. The MTT result seems to confirm this point further, as the physically-absorbed FGF group stimulated more cell growth than the dopamine immobilized group (Fig. 5). Additionally, it is worth to note that we did not use immunofluorescent staining to verify the immobilization or absorption of FGF on the surface/inside of the PXDDA film. This is due to the interruption of the auto-fluorescence of PXDDA^35^, which caused the challenge in differentiating the immunofluorescence from auto-fluorescence.

One of the most significant challenges in this area for promoting tissue regeneration is related to adequate GFs’ concentrations and gradients^47,48^. These critical factors can be controlled by modifying the surface. A simple and inexpensive method is to coat the substrates with dopamine. Dopamine can be simply deposited on various kinds of organic and inorganic surfaces to make a polydopamine film. The preliminary advantage of a polydopamine layer is related to its strong adhesion properties to almost all types of substrates, regardless of the chemical properties of surfaces^33,34^. It means that the bulk and surface structure of PXDDA lead to the efficient loading of growth factors and dopamine coating is a second step to enhance this efficacy and control. Our results show that using a polydopamine as a modification factor can be an effective method for grafting growth factors onto biomaterials surfaces in order to improve bioactivity and sustained the release in a longer term for a continuous stimulus of cell growth. So, we can consider a dopamine coating as a release controlling factor.

However, we did not investigate the controllability of different coating concentrations of dopamine on the loading efficiency and release control of the growth factors. We also did not study whether the effect of different FGF concentrations on the PXDDA discs on cell behavior. This study is a proof-of-concept to show if PXDDA with dopamine coating can effectively control the release of FGF and have a significant effect on cell morphology. More experiments on the effect of the drug-delivery system on other growth factors need to be performed to validate the robustness of this system. Also, the *in vivo* safety study of the system and its *in vivo* potential in wound healing and tissue regeneration are also needed in the next trajectory of this research.

## Conclusion

In this study, we developed growth factor-loaded PXDDA films modified using polydopamine-assisted immobilization of FGF through a simple coating process. The polydopamine coating on the PXDDA films enhanced the binding sites to growth factor FGF, and FGF bound on the PXDDA surface slowly released. *In vitro* studies showed that the FGF-polydopamine-PXDDA films have a significant role in supporting adhesion, spreading, and proliferation of fibroblast cells. Quantification of FGF on coated PXDDA films showed that increasing the amount of FGF in the treatment solution, enhanced the level of immobilized FGF. Studies with fibroblast cells also indicated initial adhesion and proliferation of cells cultured on PXDDA films modified with FGF. These results showed that polydopamine coating is a cheap, simple, and effective method for GF immobilizing onto the biomaterial surfaces, and the FGF immobilized polydopamine-PXDDA films are a promising candidate for guided tissue regeneration.

## Data Availability

The raw/processed data required to reproduce these findings cannot be shared at this time due to legal or ethical reasons.
